# Trunk Kinematics in Writhing and Fidgety Movements: A Pilot Study on Early Postural Control in Infants Using Computer Vision

**DOI:** 10.3390/bioengineering13010091

**Published:** 2026-01-13

**Authors:** Lucía Fernanda Flores-Santy, Karina Elizabeth Flores Santy, Juan Pablo Hervás-Pérez

**Affiliations:** 1 MOVS Research Group, Pontificia Universidad Católica del Ecuador, Quito 170525, Ecuador; 2 Faculty of Health Sciences–HM Hospitals, University Camilo José Cela, Urb. Villafranca del Castillo, 49, Villanueva de la Cañada, 28692 Madrid, Spain; jphervas@ucjc.edu; 3 Department of Pediatrics, Hospital Metropolitano de Quito, Quito 170509, Ecuador

**Keywords:** motor activity, psychomotor performance, child development, movement, core

## Abstract

**Background**: General Movement Assessment is a strong early predictor of adverse neurodevelopmental outcomes but remains qualitative and examiner-dependent. Quantitative, video-based kinematic analysis may complement General Movement Assessment by providing objective, scalable metrics. **Methods**: In this pilot study, a computer–vision-based pipeline was used to extract trunk center-of-mass kinematics from video recordings of spontaneous General Movements in infants under three months corrected age during the Writhing and Fidgety stage. Two measures were derived: trunk quantity of motion and movement duration. Group differences were examined using *t*-tests and effect sizes, and associations with corrected age and sex were explored with correlation analyses. **Results**: Writhing Movements were substantially longer than Fidgety Movements, with a large effect size, whereas trunk quantity of motion did not differ meaningfully between movement types. Correlations between corrected age and both the quantity of motion and duration were small and imprecise. Sex did not moderate duration changes, but trunk motion showed a significant age–sex interaction effect. **Conclusions**: Video-based extraction of trunk kinematics is feasible in early infancy and reveals robust differences in GMs type duration between Writhing and Fidgety Movements. Larger longitudinal studies are needed to clarify the value of these measures as early quantitative markers of postural control and neuromotor development.

## 1. Introduction

Trunk control plays a foundational role in early neurodevelopment, providing the postural stability necessary for upper and lower limb function. This control emerges through the integration of cerebellar, spinal, and cortical structures, and it improves as myelination and plasticity processes mature in response to environmental stimuli [1,2,3]. During the first year of life, trunk stability supports the acquisition of gross motor milestones such as rolling, sitting, crawling, standing, and walking [4,5]. To maintain balance during spontaneous movements, the center-of-mass (CoM) of the trunk must remain within the base of support, which dynamically adjusts as development progresses [6]. When postural equilibrium is challenged, infants rely on compensatory mechanisms involving trunk and limb interactions to stabilize their bodies [7,8].

In supine positions, movements of the upper and lower limbs influence trunk displacement and stability. Reaching, lifting the pelvis, and grasping the knees or feet involve anticipatory postural adjustments and abdominal activation that contribute to early trunk control [3,9]. These biomechanical interactions are essential for developing refined motor coordination and preparing the infant for upright positions.

Despite its importance, trunk kinematics during early infancy is rarely quantified in clinical practice. Traditional motor assessments often focus on milestone achievement and muscle tone but neglect the dynamic properties of spontaneous movement [10,11], The General Movements Assessment (GMA), developed by Prechtl, offers a qualitative analysis of spontaneous movement patterns in infants from fetal life to 20 weeks post-term [12]. General Movements (GMs) are complex, variable sequences involving the whole body and are classified into Writhing Movements (WM) and Fidgety Movements (FM), depending on the infant’s age and motor repertoire [13,14,15].

WM appear between 6 and 9 weeks post-term and are characterized by slow, elliptical motions involving the neck, trunk, and limbs. FM emerge around 9 to 20 weeks and are defined by smaller movements of moderate speed and amplitude, including trunk rotation and axial rolling [12,16]. The presence or absence of FM has been shown to be a strong predictor of later neurodevelopmental disorders such as cerebral palsy, with high sensitivity and specificity [15,17,18]. Nevertheless, GMA requires specialized training, limiting its accessibility—especially in low-resource settings.

To address this gap, recent studies have explored the use of computer vision and machine learning to quantify infant motor patterns [17,18,19,20]. Most existing tools rely on complex or costly equipment, such as accelerometers or 3D motion capture, which restrict their clinical applicability [21,22]. There is a growing need for accessible, low-cost technologies capable of quantifying key kinematic variables—such as movement duration and displacement of the trunk’s CoM—during spontaneous GMs.

This pilot study aims to assess whether a video-based computer vision technique can quantitatively characterize trunk CoM kinematics during whole-body and fine motor movements in infants under three months old. The analysis focuses on (1) the Quantity of Motion (QoM) of the trunk CoM and (2) the duration of GMs episodes, with the goal of identifying their potential as early quantitative markers of neuromotor development.

## 2. Materials and Methods

### 2.1. Study Design and Ethical Approval

An observational, descriptive, cross-sectional study was conducted with approval from the Human Research Ethics Committee of the Pontifical Catholic University of Ecuador (Code 2019-61-EO). All parents provided written informed consent prior to participation. A total of 32 infants under 3 months of corrected age were included through a non-probabilistic, purposive sampling strategy. Inclusion criteria were: no history of neurological risk and an APGAR score > 7 at 1 and 5 min post-birth [23]. Exclusion criteria included respiratory complications during delivery or prior diagnoses of respiratory, genetic, or motor disorders.

The current study served as a pilot and feasibility assessment to examine the potential for extracting quantitative trunk kinematic measurements from standard clinical videos of GMs. The sample size was deliberately kept small, aligning with prior feasibility research in early infant motor analysis utilizing computer vision techniques. The analysis focused on individual GMs episodes rather than the infants themselves, resulting in 32 participants’ movements available for analysis, 12 WM and 20 FM. This approach underscores the exploratory objective of the study, emphasizing methodological validation and effect size estimation over broader population generalization.

### 2.2. Video Acquisition Protocol

Recordings were conducted following the standardized guidelines of Prechtl’s GMA [12,16]. All procedures took place in a controlled clinical setting maintained at 24–26 °C, with soft indirect lighting to minimize glare and abrupt contrast changes. Background noise and external stimuli were minimized to preserve spontaneous motor behavior.

Infants were placed supine on a firm, warm surface, fully awake, undressed to the diaper, and in a calm, alert state. A caregiver and an evaluator remained nearby to ensure comfort without interfering with spontaneous movements.

Video recordings were captured using a 23 MP, 24 mm wide-angle lens digital camera (1080p, 30 fps, HDR enabled) mounted on a fixed tripod positioned 1 m above the infant, providing a perpendicular, top-down perspective.

The camera’s height, angle, and position were maintained consistently across all sessions to minimize geometric variability. Additionally, planar pixel-to-metric calibration using a measurement bar enabled metric scaling within each recording, although comprehensive camera intrinsic calibration was not conducted.

Given the use of a 24 mm wide-angle lens, some degree of radial distortion may be present; however, its impact was minimized by restricting the analysis to a trunk-centered region of interest and by using planar scaling based on the local calibration object.

For pixel-to-metric calibration, a rigid measurement bar with millimeter-scale markings was placed on the recording surface within the camera’s field of view in each video. This reference object was positioned on the same plane as the infant’s trunk and remained visible during acquisition, enabling the calculation of a planar conversion factor (pixels per millimeter) for each recording. This calibration process allowed for the subsequent conversion of trunk CoM kinematic measures from image units to metric units during post-processing.

To optimize video quality for computer vision analysis, ambient lighting was controlled to prevent shadows or reflections. No zoom or digital stabilization was used, avoiding artificial motion artifacts. Prior to each session, the mat and background were inspected to ensure a clear visual contrast with the infant’s body, supporting reliable trunk segmentation and center-of-mass detection.

Each session lasted 30 min, following a standardized clinical–engineering protocol aimed at capturing natural spontaneous movement while providing high-quality input for kinematic processing.

### 2.3. General Movements Procedures and Classification

All infants underwent a standardized GMA recording protocol conducted in a controlled clinical environment specifically designed for infant neurodevelopmental evaluations. A certified pediatric healthcare professional trained in Prechtl’s method supervised all procedures to ensure adherence to international GMA guidelines. Each 30 min recording was independently reviewed by two pediatric health professionals formally certified in the Prechtl GMA through the General Movement Trust training program. Both evaluators had more than 10 years of clinical experience in neonatal and infant motor assessment and were blinded to the infants’ clinical histories. Using Prechtl’s qualitative criteria, evaluators classified spontaneous motor behavior into WM and FM categories. Infants exhibiting abnormal GMs patterns—such as poor repertoire, cramped-synchronized, or chaotic movements—were excluded from the study to prevent confounding motor phenotypes from affecting the kinematic analysis.

Interrater agreement for WM/FM classification was assessed using Fleiss’ Kappa coefficient in accordance with established standards for categorical motor evaluations. When disagreements occurred, a senior pediatric neurologist specializing in early neuromotor development acted as an adjudicator. This neurologist, who was not involved in the initial assessments, provided a consensus resolution without altering the original independent ratings.

### 2.4. Computer Vision Pipeline 

All segmented video sequences were analyzed using a custom computer vision pipeline developed in MATLAB^®^ v9.12 (MathWorks, Natick, MA, USA). The purpose of the proposed computer vision pipeline is to extract biomechanically interpretable kinematic features from clinical video recordings, rather than to develop or validate a predictive or classification model. This pipeline relied on two well-established toolboxes: MatlabPyrTools^®^ for multiscale image processing and contrast enhancement, and MoCap Toolbox^®^ for spatiotemporal analysis of kinematic coordinates. The primary aim of the pipeline was to extract quantitative descriptors of trunk CoM displacement during GMs.

#### 2.4.1. Preprocessing and Frame Standardization

To ensure consistent temporal resolution and enable precise comparisons across recordings, all GM segments were uniformly resampled via temporal interpolation. Each sequence was adjusted to a standardized rate of 400 frames per second (fps), establishing a consistent temporal framework for subsequent frame-by-frame kinematic analysis. The videos were then converted to a standardized format and visually examined to verify correct segmentation of the target GMs type episode.

For each sequence, a region of interest (ROI) covering the infant’s trunk was manually segmented. This ROI-focused approach concentrated processing on the relevant anatomical area for CoM estimation, while reducing background interference and extraneous motion.

The pixel-to-metric conversion factor was determined by manually identifying the known length of the measurement bar within the image plane and calculating the corresponding pixel distance. This factor was then applied uniformly to the trunk CoM trajectories within the region of interest, assuming planar motion relative to the recording surface. This methodology is commonly used in 2D video-based biomechanical analyses when full camera intrinsic calibration is unavailable.

#### 2.4.2. Image Enhancement and Silhouette Extraction

Multiscale filtering utilizing MatlabPyrTools^®^ within the ROI was implemented to improve contrast and highlight the structural features of the trunk region. This process enhanced the robustness of subsequent silhouette detection despite natural variations in illumination, clothing texture, and background.

After enhancement, an intensity-based thresholding method combined with standard morphological operations such as opening and closing was used to isolate the trunk silhouette within the ROI. These image processing techniques helped reduce noise, eliminate small spurious regions, and produce a clean binary silhouette suitable for centroid-based analysis. No manual annotation of anatomical landmarks was performed; all CoM estimates were obtained solely from the binary trunk silhouette.

#### 2.4.3. CoM Estimation

For each frame, the trunk CoM coordinates were estimated utilizing functions from the MoCap Toolbox^®^, which calculate the geometric centroid of the segmented trunk silhouette. This method assumes an approximately uniform mass distribution within the trunk region, a common and practically validated approximation in video-based infant motor analysis.

Consequently, for each time frame *t*, the CoM position was designated as the centroid of the binary silhouette within the ROI. The series of frame-by-frame CoM coordinates across the resampled video constituted a two-dimensional displacement trajectory, depicting trunk movement over time in the image plane.

#### 2.4.4. QoM and Kinematic Feature Extraction

The primary kinematic variable obtained was the QoM of the trunk CoM. QoM was defined as the sum of the absolute frame-to-frame displacements of the CoM throughout the episode:

$$QoM=\sum_{t=1}^{N-1}∥CoM(t+1)-CoM(t)∥$$

where *N* is the total number of frames in the resampled sequence and ‖⋅‖ denotes the Euclidean distance in the image plane.

To account for variations in episode length, QoM values were normalized relative to GM duration, resulting in a duration-normalized QoM metric. Additional kinematic parameters included episode duration (s) and mean displacement rate, calculated as the total CoM path length divided by episode duration (measured in pixels per second). All variables were determined separately for WM and FM segments.

#### 2.4.5. Quality Control and Reproducibility

Several procedures were implemented to ensure the reliability and reproducibility of the extracted kinematic measures. First, random sample frames from each episode were visually inspected to verify the integrity of the extracted silhouette and the correct localization of the trunk. Second, sequences were screened for artifacts such as transient loss of the silhouette, and obvious artifacts were either corrected or excluded. Third, camera geometry was maintained consistently across infants, with a fixed overhead viewpoint at approximately 1 m above the infant, to minimize variation in perspective and scaling. Finally, ROI selection was standardized and performed by the same trained evaluator for all recordings.

This pipeline was intentionally designed to emphasize interpretability, reproducibility, and clinical practicality over algorithmic complexity. By using silhouette-based trunk segmentation and centroid estimation, the approach eliminates the need for anatomical landmark annotation, wearable sensors, or deep learning architectures, thereby enabling transparent interpretation of kinematic outputs and supporting potential clinical implementation in low-resource environments.

### 2.5. Statistical Analysis

All statistical analyses were performed using IBM SPSS Statistics version 28.0 (IBM Corp., Armonk, NY, USA). An a priori power analysis suggested that at least 32 participants were needed to detect moderate between-group differences (Cohen’s d = 0.50) at an alpha level of 0.05 with 80% power. Since the study included exactly 32 infants, the sample size was deemed sufficient for analyzing the primary comparison of movement duration between WM and FM [12,24,25]. Applying a Bonferroni correction for the two primary outcomes may slightly reduce the statistical power, which is acceptable within the context of a pilot study [26].

All statistical analyses were conducted at the episode level. Each entry in the dataset represented a single GMs, including variables such as movement type (FM and WM), corrected age at the time of recording, trunk CoM QoM, sex, and the duration of the GMs.

Continuous variables, including corrected age, QoM, and GMs type duration, were initially summarized using descriptive statistics. For the overall sample and by movement type, the mean, standard deviation (SD), median, interquartile range (IQR), minimum, and maximum values were reported. Distributions of QoM and episode duration were visually assessed through histograms and boxplots, while potential differences between movement types were further examined with stratified boxplots.

To compare kinematic measures between FM and WM, movement type was treated as a binary grouping factor. Differences in QoM and episode duration between groups were assessed using independent-samples *t*-tests. When there was concern about unequal variances, Welch’s *t*-test was employed. Standardized mean differences were quantified with Cohen’s d, calculated using the pooled standard deviation. For each effect size, 95% confidence intervals were derived based on the sampling distribution of Cohen’s d. These effect sizes were interpreted alongside *p*-values to highlight the magnitude and precision of group differences.

In addition to standardized effect sizes, unstandardized mean differences between WM and FM were calculated for all continuous outcomes. Mean differences are reported together with their 95% confidence intervals, estimated using Welch’s method when variance heterogeneity was present. Reporting both standardized and unstandardized effects improves interpretability and facilitates comparison across studies.

To explore developmental trends, we analyzed the relationships between corrected age and kinematic outcomes, specifically QoM and episode duration. For each outcome, Pearson’s product–moment correlation coefficient (r) was calculated to assess linear associations, while Spearman’s rank correlation coefficient (ρ) evaluated monotonic (rank-based) associations. Correlations were initially computed across all episodes, and subsequently within each movement category, to identify potential category-specific patterns.

Confidence intervals for Pearson correlation coefficients were derived using Fisher’s z-transformation with normal-theory limits. For Spearman rank correlations, 95% confidence intervals were estimated using a nonparametric bootstrap approach (bias-corrected and accelerated, BCa, 10,000 resamples), which is more appropriate for small sample sizes and does not rely on large-sample asymptotic assumptions. Spearman correlations computed within very small subgroups were interpreted descriptively, with emphasis on effect direction and magnitude rather than statistical significance.

To determine whether sex moderates the association between corrected age and kinematic outcomes, linear regression models including an age × sex interaction term (Outcome ~ Age + Sex + Age × Sex) were employed. The interaction effects were assessed through regression coefficients, 95% confidence intervals, and *p*-values. As a complementary sensitivity analysis, Bayesian linear models were utilized to calculate Bayes factors (BF10), comparing models with and without the interaction term to estimate the evidence for sex-specific moderation.

All analyses were conducted and interpreted with an understanding of the pilot nature and the limited sample size of this study. Accordingly, priority was given to descriptive statistics, effect sizes, and confidence intervals rather than formal hypothesis testing or strict *p*-value thresholds.

## 3. Results

### 3.1. Sample Characteristics

A total of 32 infants with a corrected age of less than 3 months were included in the GM analysis. Each observation documented the corrected age (weeks), sex, movement type, trunk CoM QoM, and episode duration (s).

**Figure 1:**
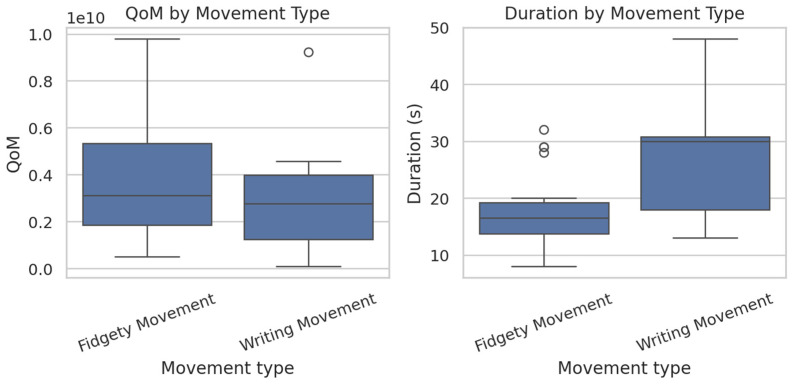
QoM and GMs type duration by movement category. Boxes represent the interquartile range (IQR), horizontal lines the median, and whiskers 1.5 × IQR.

FM were observed slightly more frequently than WM (20 versus 12), and male infants were more prevalent than female infants (20 males versus 12 females). Descriptive statistics for corrected age, QoM, and episode duration are summarized in Table 1. Variability in QoM and episode duration was observed, reflecting the exploratory and pilot nature of this study (Figure 1).

### 3.2. Comparison Between FM and WM Groups

Group comparisons between FM and WM focused on two primary outcomes: the QoM of the trunk CoM and the duration of GMs. For QoM, WM showed a lower mean than FM (2.94 × 10^9^ vs. 3.99 × 10^9^), with a mean difference (WM–FM) of −1.05 × 10^9^. An independent-samples *t*-test with Welch correction indicated that this contrast was not statistically significant (Welch’s *t* = −1.05, *p* = 0.30), and the 95% confidence interval for the mean difference (−3.05 × 10^9^ to 0.96 × 10^9^) included zero. The standardized mean difference (Cohen’s d = −0.36) was small to moderate, suggesting no clear evidence for a systematic difference in trunk QoM between WM and FM in this pilot sample.

In contrast, GMs type duration differed markedly between movement categories. WM were longer than FM (26.83 s vs. 17.90 s), with a mean difference (WM–FM) of 8.93 s. This difference was statistically significant (Welch’s *t* = 2.68, *p* = 0.02), and the 95% CI for the mean difference (2.10 to 15.80 s) did not include zero. The corresponding effect size was large (Cohen’s d = 1.08), indicating that movement type is strongly associated with GMs length in this dataset (Table 2).

### 3.3. Association of Corrected Age with QoM and Duration

Correlations between corrected age and kinematic measures are summarized in Table 3. Across all GMs types, corrected age displayed minimal association with trunk CoM QoM, as indicated by both Pearson correlation (r = 0.09, 95% CI −0.26 to 0.43, *p* = 0.61) and Spearman correlation (ρ = 0.02, 95% CI −0.33 to 0.37, *p* = 0.91). In contrast, corrected age showed a moderate negative association with episode duration. Pearson’s r suggested a non-significant trend (r = −0.32, 95% CI −0.60 to 0.04, *p* = 0.078), while Spearman’s ρ demonstrated a statistically significant monotonic relationship (ρ = −0.39, 95% CI −0.65 to −0.05, *p* = 0.027), indicating that older infants tended to have shorter GMs.

When stratified by movement category, the correlations between corrected age and QoM or duration remained small and were not statistically significant for both FM and WM (all |r| ≤ 0.32, all |ρ| ≤ 0.39, all *p* ≥ 0.25). Wide confidence intervals were observed, reflecting limited subgroup sample sizes.

### 3.4. Age × Sex Moderation of GMs Duration and Trunk CoM Quantity of Movement

To examine whether sex moderated age-related changes in FM, we restricted the analysis to GMs classified as FM (n = 20; 10 males, 10 females). For each participant, we considered corrected age, sex, GMs type duration (s), and the trunk CoM QoM.

#### 3.4.1. GMs Type Duration

In sex-stratified correlations, GMs type duration was weakly negatively associated with corrected age in males (r ≈ −0.29) and weakly positively associated in females (r ≈ 0.25). To formally test whether these apparent differences reflected a true sex moderation, we fitted a linear model including an Age × Sex interaction (Duration ~ Age + Sex + Age × Sex).

The interaction term was not statistically significant (β_age × sex = −0.99 s/week, *p* ≈ 0.29), and a Bayesian Information Criterion (BIC)-based approximation to the Bayes factor indicated that the model without the interaction was slightly favored over the interaction model (BF_10_ ≈ 0.45 for interaction vs. no interaction). Thus, despite opposite-signed correlations in males and females, the data do not provide compelling evidence that sex moderates the association between age and GMs type duration, likely reflecting the limited precision associated with the small subgroup sizes (10 GMs per sex).

#### 3.4.2. Trunk CoM QoM

In contrast, the relationship between age and trunk CoM QoM showed a clear sex-dependent pattern. Among males, QoM tended to decrease with age (r ≈ −0.63), whereas among females it increased with age (r ≈ 0.65). A linear model with an Age × Sex interaction (QoM ~ Age + Sex + Age × Sex) confirmed this moderation effect: the interaction term was statistically significant (β_age × sex ≈ −1.05 × 10^9^ a.u./week, *p* ≈ 0.004).

**Figure 2:**
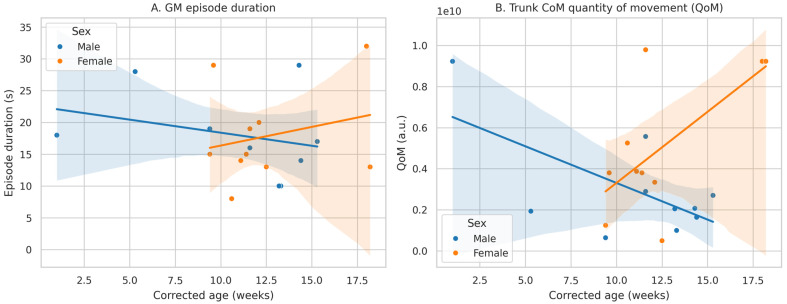
Age–sex moderation of FM duration and trunk CoM QoM. (**A**) Scatterplot and sex-specific regression lines for the association between corrected age and FM GMs type duration. Although correlations differ in sign between males and females, the Age × Sex interaction is not statistically supported. (**B**) Scatterplot and sex-specific regression lines for the association between corrected age and trunk CoM QoM during GMs. The Age × Sex interaction is statistically significant and a BIC-based Bayes factor strongly favors the interaction model, indicating that QoM increases with age in females but decreases with age in males.

A BIC-based Bayes factor approximation strongly favored the interaction model over the additive model without the interaction (BF_10_ ≈ 46.4), providing robust evidence that the age–QoM association differs by sex. Specifically, as illustrated in Figure 2, the regression lines for males and females diverge with age, with male infants showing a progressive reduction in trunk CoM QoM and female infants showing an increase across the studied corrected-age range.

## 4. Discussion

This pilot study examined whether a video-based computer vision approach can quantitatively analyze trunk CoM kinematics during WM and FM in infants under three months corrected age, emphasizing trunk CoM QoM and GMs type duration as potential early indicators of postural control and neuromotor development. The results demonstrate the feasibility of markerless, video-based tracking of trunk motion within this age group and suggest that GMs type duration, rather than a global QoM index, most clearly differentiates between WM and FM patterns.

In comparison with state-of-the-art approaches that predominantly employ machine learning or deep neural networks to classify GMs or predict cerebral palsy risk, the present study adopts a complementary bioengineering perspective. While recent models primarily emphasize classification accuracy using high-dimensional motion descriptors or pose-estimation outputs, our approach focuses on biomechanically meaningful trunk kinematic variables that can be directly interpreted in relation to early postural control. Rather than competing with existing AI-based frameworks, trunk CoM metrics may enrich multimodal assessment strategies by providing clinically interpretable descriptors that align with established neurodevelopmental theory.

The first key result is that WM were substantially longer than FM, with a large standardized mean difference. This observation is consistent with classical descriptions of GM patterns: WM in early term and preterm infants are typically larger-amplitude, whole-body movements that can be sustained over longer episodes, whereas FM are small-amplitude, continuously present but relatively brief, variable movements emerging around 9–16 weeks post-term [27,28,29]. Our quantitative duration findings align with this qualitative clinical characterization and suggest that GMs type duration may capture an aspect of the temporal organization of early motor behavior that distinguishes movement states at the trunk level. In line with the strong prognostic value of abnormal or absent FM for CP and other adverse outcomes [30,31,32], the ability to quantify how long different movement states are expressed may ultimately enhance risk stratification, although this requires outcome data that were beyond the scope of this pilot.

In contrast, trunk CoM QoM did not differ significantly between WM and FM, and the effect size was small and imprecise. This might indicate that, at the level of gross trunk displacement, both movement categories involve comparable overall activity, even if they differ in complexity, fluency, and segmental coordination, which human observers perceive during GMA [28,31,33]. Prior quantitative studies have similarly shown that simple movement amplitude or displacement metrics may be less discriminative than measures capturing spatial–temporal complexity, variability, or inter-limb coordination when distinguishing typical from atypical movement patterns [24,33,34,35]. Our results therefore suggest that a single scalar QoM index of trunk CoM may be too coarse to reflect the qualitative richness of FM versus WM. Future work may need to augment QoM with additional kinematic features such as entropy, frequency content, intra-episode variability, or multi-segment coordination measures, as explored in other infant-movement studies [36,37,38,39].

The modest and imprecise associations we found between corrected age and both trunk CoM QoM and movement duration are not unexpected in a small pilot sample and a narrow age window. Developmental studies have shown that early motor trajectories in the first months of life are characterized by substantial inter-individual variability and non-linear change, especially around the transition from WM to FM and the emergence of more purposeful motor behaviors [27,28,40]. Moreover, the prognostic power of GMA stems more from the quality and pattern of movements than from simple linear relations between age and single kinematic parameters [31,32,33]. Our correlation findings should be regarded as preliminary and underpowered, rather than as evidence against meaningful developmental trends. To accurately model age-related changes in trunk kinematics and differentiate developmental variability from early pathological markers, larger samples with wider age ranges and repeated assessments will be necessary [30,32,40].

Sex-stratified analyses revealed potential differences in the association between corrected age and trunk kinematic outcomes; however, these patterns differed depending on the specific variable examined. For GMs type duration, CI remained wide and overlapping, and formal age × sex interaction testing did not provide evidence of sex-specific modulation within this pilot sample. Existing studies report modest sex differences in neurodevelopmental vulnerability, with a higher risk of adverse outcomes among preterm males [41,42], and some research has described sex-related variations in early motor trajectories [43].

In contrast, trunk CoM QoM exhibited a statistically significant age × sex interaction, with Bayesian analysis providing strong evidence in favor of sex-dependent developmental trajectories. Given the limited sample size and exploratory nature of this study, this finding should be interpreted cautiously and regarded as hypothesis-generating rather than confirmatory. Larger longitudinal studies will be required to determine whether sex-related differences in trunk kinematics represent stable developmental patterns or transient effects during early infancy [44,45].

From a methodological perspective, this study contributes to an expanding body of research employing computer vision and machine learning techniques for infant movement analysis. Multiple research groups have demonstrated that automated analysis of spontaneous movements from 2D video, using motion segmentation or pose estimation, can effectively identify infants at high risk for CP, with accuracy often comparable to or surpassing expert GMA [24,46]. These approaches predominantly focus on whole-body or limb kinematics, spatiotemporal motion descriptors, or data-driven feature extraction. Our research adds a complementary, biomechanically oriented perspective by concentrating specifically on trunk CoM as an indicator of early postural control. The trunk’s pivotal role in stabilizing the body and supporting the development of reaching, sitting, and other goal-directed behaviors later in infancy is well recognized [40,47]. By demonstrating that trunk CoM kinematics can be reliably extracted from routine video recordings and utilized to distinguish between WM and FM, this work paves the way for incorporating trunk-focused metrics into multimodal early screening protocols.

At the same time, this study has several limitations that should be considered. First, the sample size was limited and restricted to a narrow developmental window, resulting in wide confidence intervals and reduced statistical power to detect subtle age- or sex-related effects. Second, although planar calibration with a measurement bar allowed for pixel-to-metric conversion, future research would benefit from full camera intrinsic calibration to further mitigate lens distortion and enhance cross-study comparability. Lastly, relying on a single global trunk CoM descriptor may not fully represent the complexity of spontaneous movement patterns observed during GMs [28,31].

Despite these limitations, the current study aligns with the goals of the bioengineering discipline to create non-invasive, scalable, and objective methods for early neuromotor assessment. By using computer vision to analyze trunk kinematics from standard video footage, we move toward an accessible, markerless approach that can supplement expert-based GMA, particularly in environments where trained assessors are limited. Future investigations should involve multi-center, longitudinal studies that combine trunk CoM metrics with whole-body kinematics, comprehensive clinical profiles, and standardized neurodevelopmental outcomes. Such research will be essential to establish whether the differences observed in GMs type duration between WM and FM, along with more detailed trunk kinematic features, can reliably serve as early biomarkers for postural control and neuromotor development.

In summary, this pilot study illustrates that video-based computer vision techniques can quantitatively assess trunk CoM kinematics during WM and FM in early infancy. Although trunk CoM QoM showed minimal differences between movement categories, WM were significantly longer than those of FM, aligning with established qualitative descriptions of these movement types [27,28,29]. These results support the feasibility of deriving quantitative, trunk-focused metrics from routine videos and lay the groundwork for future larger bioengineering investigations aimed at integrating such measures into early screening and monitoring of neuromotor development.

## 5. Conclusions

This pilot study demonstrates the feasibility of using video-based computer vision techniques to extract quantitative trunk kinematic features from spontaneous GMs in early infancy. Using an affordable and reproducible pipeline, we quantified movement duration and trunk CoM displacement, showing that WM were significantly longer than FM (*p* = 0.02), whereas trunk QoM did not differ meaningfully between movement types. These findings suggest that temporal parameters may be more sensitive than global spatial displacement metrics for distinguishing early movement patterns. Exploratory analyses further indicated that age-related changes in trunk QoM may differ by sex, although this result should be interpreted cautiously given the pilot nature of the study and the limited sample size. Overall, these results support the feasibility of deriving trunk-focused kinematic descriptors from routine video recordings and provide a methodological foundation for future longitudinal studies aimed at integrating quantitative trunk metrics into early neuromotor assessment frameworks.

## Figures and Tables

**Table 1:** Descriptive statistics (mean ± SD) of corrected age, QoM, and movement duration for infants with FM and WM.

	N	Corrected Age Mean	Corrected Age Std	QoM Mean	QoM Std	Movement Duration (s) Mean	Movement Duration (s) Std
FM	20	11.70	3.88	3.99 × 10^9^	3.08 × 10^9^	17.9	6.80
WM	12	6.66	2.58	2.94 × 10^9^	2.49 × 10^9^	26.83	10.28

**Table 2:** Comparison of trunk CoM QoM and movement duration between WM and FM.

Variable	N WM	N FM	Mean WM	Mean FM	Mean Difference (WM–FM)	95% CI (Mean Diff.)	Welch *t*	*p* Value	Cohen d
QoM	12	20	2.94 × 10^9^	3.99 × 10^9^	−1.05 × 10^9^	−3.05 × 10^9^–0.96 × 10^9^	−1.05	0.30	−0.36
Movement Duration (s)	12	20	26.83	17.9	8.93	2.10–15.80	2.68	0.02	1.08

WM, writhing movements; FM, fidgety movements. Values are means for each movement category. Mean Difference is calculated as WM–FM. Welch’s *t*-test was used to compare WM and FM due to unequal sample sizes. Cohen’s d represents the standardized mean difference. For trunk CoM QoM, WM showed a lower mean than FM (Mean Difference −1.05 × 10^9^; 95% CI −3.05 × 10^9^ to 0.96 × 10^9^), but this difference was not statistically significant. In contrast, WM were substantially longer than FM (Mean Difference 8.93 s; 95% CI 2.10 to 15.80 s), indicating a statistically significant and large effect on movement duration.

**Table 3:** Associations between corrected age and kinematic measures of GMs.

Group	Outcome	Pearson r (95% CI), *p*	Spearman ρ (95% CI), *p*
Overall (N = 32)	QoM	0.09 (−0.26 to 0.43), *p* = 0.61	0.02 (−0.33 to 0.37), *p* = 0.91
Overall (N = 32)	Duration (s)	−0.32 (−0.60 to 0.04), *p* = 0.078	−0.39 (−0.65 to −0.05), *p* = 0.027
Fidgety (N = 20)	QoM	0.04 (−0.41 to 0.48), *p* = 0.85	0.03 (−0.42 to 0.47), *p* = 0.90
Fidgety (N = 20)	Duration (s)	−0.05 (−0.48 to 0.40), *p* = 0.83	−0.14 (−0.55 to 0.33), *p* = 0.56
Writhing (N = 12)	QoM	−0.21 (−0.70 to 0.42), *p* = 0.52	−0.15 (−0.66 to 0.47), *p* = 0.65
Writhing (N = 12)	Duration (s)	−0.06 (−0.61 to 0.53), *p* = 0.86	−0.36 (−0.77 to 0.27), *p* = 0.25

QoM of the trunk CoM; Duration (s), duration of the GMs episode in seconds. Pearson r quantifies linear correlation; Spearman ρ quantifies rank-based (monotonic) correlation. 95% CI, 95% confidence interval. FM and WM refer to the qualitative movement classification of the recorded GMs.

## Data Availability

The dataset generated and analyzed during the current study is publicly available in the Figshare repository, at: https://doi.org/10.6084/m9.figshare.28175012.v1.

## Abbreviations

The following abbreviations are used in this manuscript:

GMs	General Movements
GMA	General Movements Assessment
FM	Fidgety Movements
WM	Writhing Movements
QoM	Quantity of movement
CoM	Center of mass
